# Effects of ergotamine on the central nervous system using untargeted metabolomics analysis in a mouse model

**DOI:** 10.1038/s41598-021-98870-4

**Published:** 2021-10-01

**Authors:** Priyanka Reddy, Delphine Vincent, Joanne Hemsworth, Vilnis Ezernieks, Kathryn Guthridge, German C. Spangenberg, Simone J. Rochfort

**Affiliations:** 1 Agriculture Victoria, AgriBio, Centre for AgriBioscience, Bundoora, Victoria 3083 Australia; 2 School of Applied Systems Biology, La Trobe University, Bundoora, Victoria 3083 Australia

**Keywords:** Biochemistry, Neuroscience

## Abstract

The ergot alkaloid ergotamine is produced by *Claviceps purpurea,* a parasitic fungus that commonly infects crops and pastures of high agricultural and economic importance. In humans and livestock, symptoms of ergotism include necrosis and gangrene, high blood pressure, heart rate, thermoregulatory dysfunction and hallucinations. However, ergotamine is also used in pharmaceutical applications to treat migraines and stop post-partum hemorrhage*.* To define its effects, metabolomic profiling of the brain was undertaken to determine pathways perturbed by ergotamine treatment. Metabolomic profiling identified the brainstem and cerebral cortex as regions with greatest variation. In the brainstem, dysregulation of the neurotransmitter epinephrine, and the psychoactive compound 2-arachidonylglycerol was identified. In the cerebral cortex, energy related metabolites isobutyryl-L-carnitine and S-3-oxodecanoyl cysteamine were affected and concentrations of adenylosuccinate, a metabolite associated with mental retardation, were higher. This study demonstrates, for the first time, key metabolomic pathways involved in the behavioural and physiological dysfunction of ergot alkaloid intoxicated animals.

## Introduction

Ergot alkaloids, the causative agents of ergotism, are commonly produced by fungi from the Clavicipitaceae family. In humans, symptoms of ergotism include gangrene, convulsions and hallucinations^1^. However, tightly controlled dosages of the ergot alkaloid, ergotamine is successfully applied in pharmaceutical applications^2^. *Claviceps purpurea* (Fries ex Fries) Tulasne*,* a commonly known parasitic fungus, that produces ergotamine, infects cereals and forage grasses with a wide host range of more than 400 plant species^3^. These include economically significant crops, such as rye, wheat and barley and temperate grasses such as tall fescue. The temperate grasses tall fescue and perennial ryegrass can also be infected with endophytic fungi of the *Epichloë* species which produces the major ergot alkaloid ergovaline. The asexual nonpathogenic species, in particular *E. festucae* var. *lolii* and *E. coenophiala,* are commonly utilised in pasture-based agriculture as they enhance disease and insect pest control and thus improve pasture performance^3–6^. However, ergovaline has well documented effects on animal welfare, including poor weight gain from reduced feed intake, decreased fertility, and poor milk production^7^.

Host plant-Clavicipitaceae fungi associations lead to production of ergot alkaloids that are known to cause ergotism in humans and other mammals who consume contaminated grains or pasture^3^. The most potent and abundant of the ergopeptides produced by *C. purpurea* and *Epichloë* sp. are ergotamine and ergovaline respectively. The physiological effects observed in animals exposed to ergotamine or ergovaline are comparable in intensity and duration. These include effects on blood pressure, heart rate and thermoregulatory dysfunction^8–10^. Furthermore, in vitro studies show the most potent contractile responses of veins and arteries occur in response to these two mycotoxins when compared to other ergopeptides produced by the fungi^11–17^.

Ergopeptides, such as ergotamine and ergovaline, are the most structurally complex ergot alkaloids, comprising D-lysergic acid linked via an amide bond to a three-membered peptide^7^. The alkaloids vary only in the composition of the peptide i.e. L-alanine, L-phenylalanine and L-proline in ergotamine and L-alanine, L-valine and L-proline in ergovaline.

Studies on the ergopeptides show they cause physiological dysfunction in respiratory, thermoregulatory, cardiac and vasomotor function as demonstrated in animal bioassays investigating the effects of ergovaline and ergotamine^7–9,18^. However, the simple ergoamide, ergine, with only a basic D-lysergic acid structure attached to an amide, is reported to possess psychotropic properties^19^.

Consumption of contaminated rye related products such as bread frequently caused ergotism epidemics throughout the Middle Ages^3^. Despite the major impact of these outbreaks, there are also historical reports of medicinal uses as well. For example, midwives used the ergot sclerotia to accelerate childbirth or initiate abortion in the sixteenth century. Later in the nineteenth century the ergot alkaloids were used to prevent excessive bleeding in childbirth and is used even today in developing countries, which contributes to the high rates of maternal mortalities^3^. Furthermore, in the latter half of the nineteenth century, controlled dosages of ergot alkaloids such as ergotamine were effectively used to treat severe and acute migraines^2,20^. In more recent times ergot alkaloids have been modified for pharmaceutical use and these include compounds such as pergolide and cabergoline which are used for neurological disorders including Parkinsons disease and Restless leg syndrome^21^.

Naturally occurring ergot alkaloids exert their effects on dopamine, serotonin and adrenaline neurotransmitter receptor sites, acting as partial agonists and in some cases as antagonists. Studies conducted on ergot alkaloid producing fungus, *E. coenophialum,* an endophyte associated with tall fescue grass (*Lolium arundinaceum*), have shown to disrupt brain dopaminergic and serotonergic mechanisms in the central nervous system^22,23^ as well as inhibit activity of Na+/K+ ATPase, essential for maintenance of neuronal membrane potential^24^.

Despite studies evaluating the effect of mycotoxins on the central nervous system, the mode of action of the ergot alkaloid family of toxins within the brain is still largely unknown. To address this, we investigate the effects of ergotamine on the metabolomic profile of the mouse brain and identify key metabolites associated with the physiological and behavioural effects elicited by animals exposed to ergot alkaloids.

## Results

**Figure 1:**
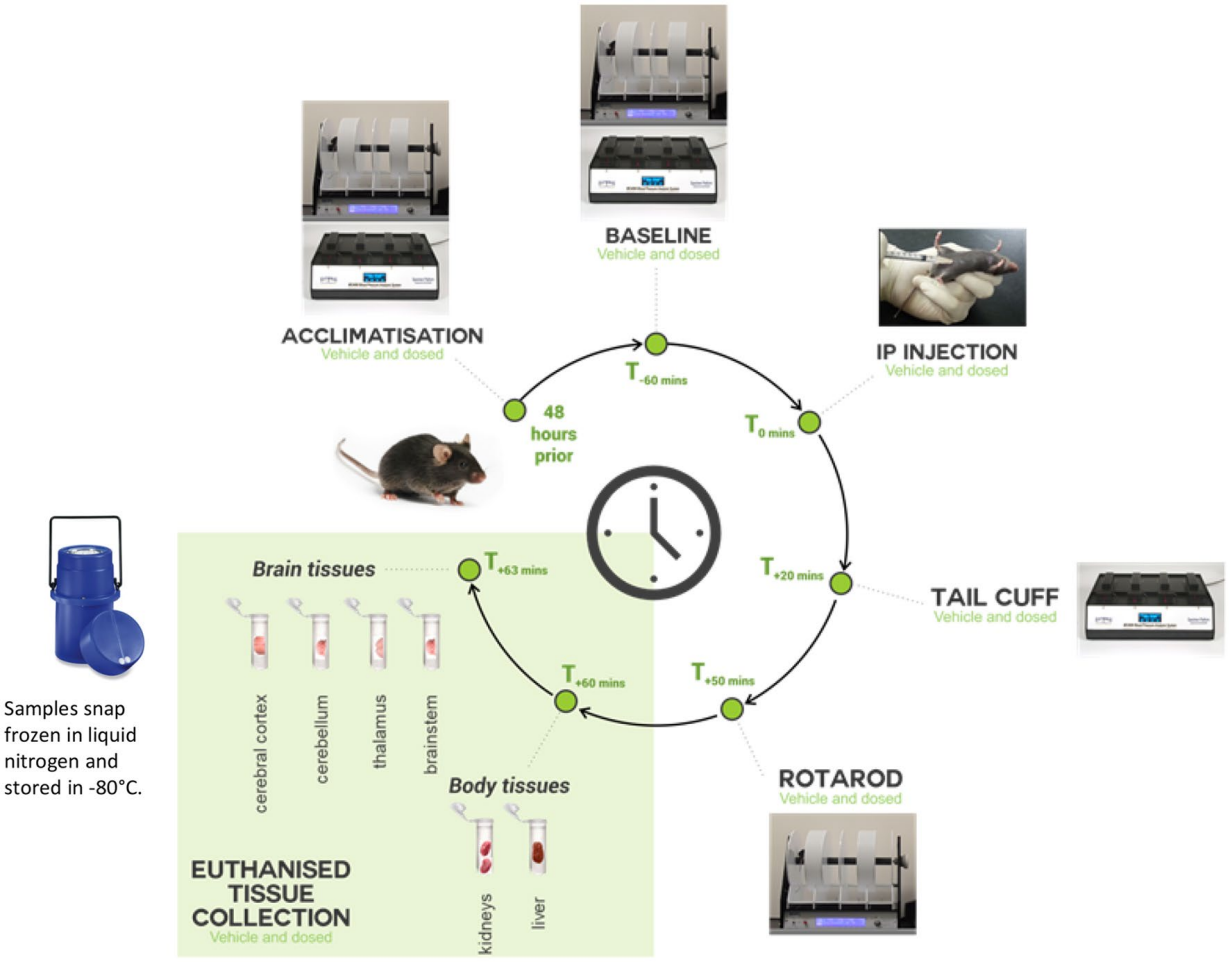
Experimental design of mouse bioassay for ergot alkaloid intoxication.

To investigate the effects on the central nervous system, various brain tissues (cerebral cortex, brainstem, thalamus and cerebellum) were harvested from vehicle control (ET^VEH^)_,_ ergotamine high dose (ET^HIGH^) and ergotamine low dose (ET^LOW^) treated mice 1 h post exposure (Fig. 1) and the metabolite extracts were analysed using ESI+ Liquid Chromatography-Mass Spectrometry (LCMS).

**Figure 2:**
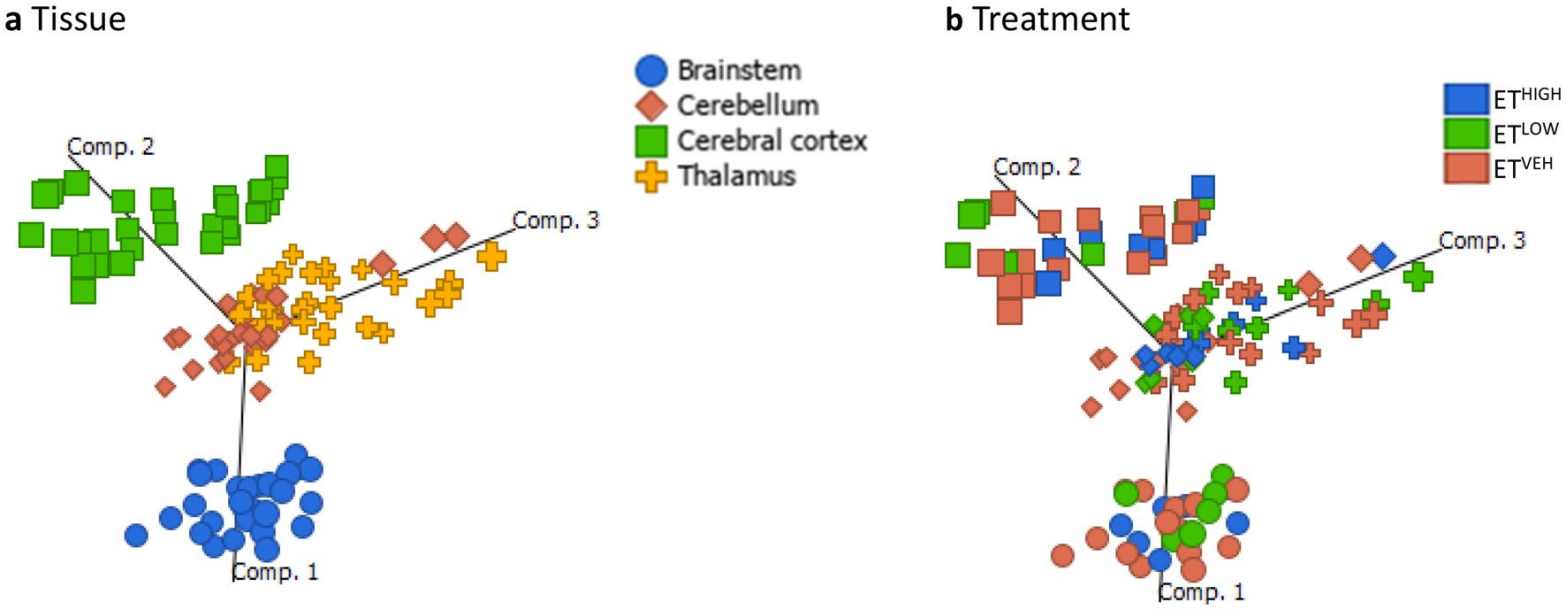
PCA scores plots of ESI + UHPLC-HRMS data coloured by (**a**) brain tissues: brainstem, cerebellum, cerebral cortex and thalamus and (**b**) treatments: ET^HIGH^ (blue; 0.05 mg/kg; n = 8), ET^LOW^ (green; 0.025 mg/kg; n = 6) and ET^VEH^ (orange; 1% lactic acid; n = 8).

PCA plots of untreated and treated brain tissues revealed separation of the four regions, with some overlap in the thalamus and cerebellum regions of the brain (Fig. 2). It is not unexpected that the metabolome of the individual regions of the brain are distinct due to their inherently unique functions. All subsequent analyses were conducted separately for each brain region.

**Figure 3:**
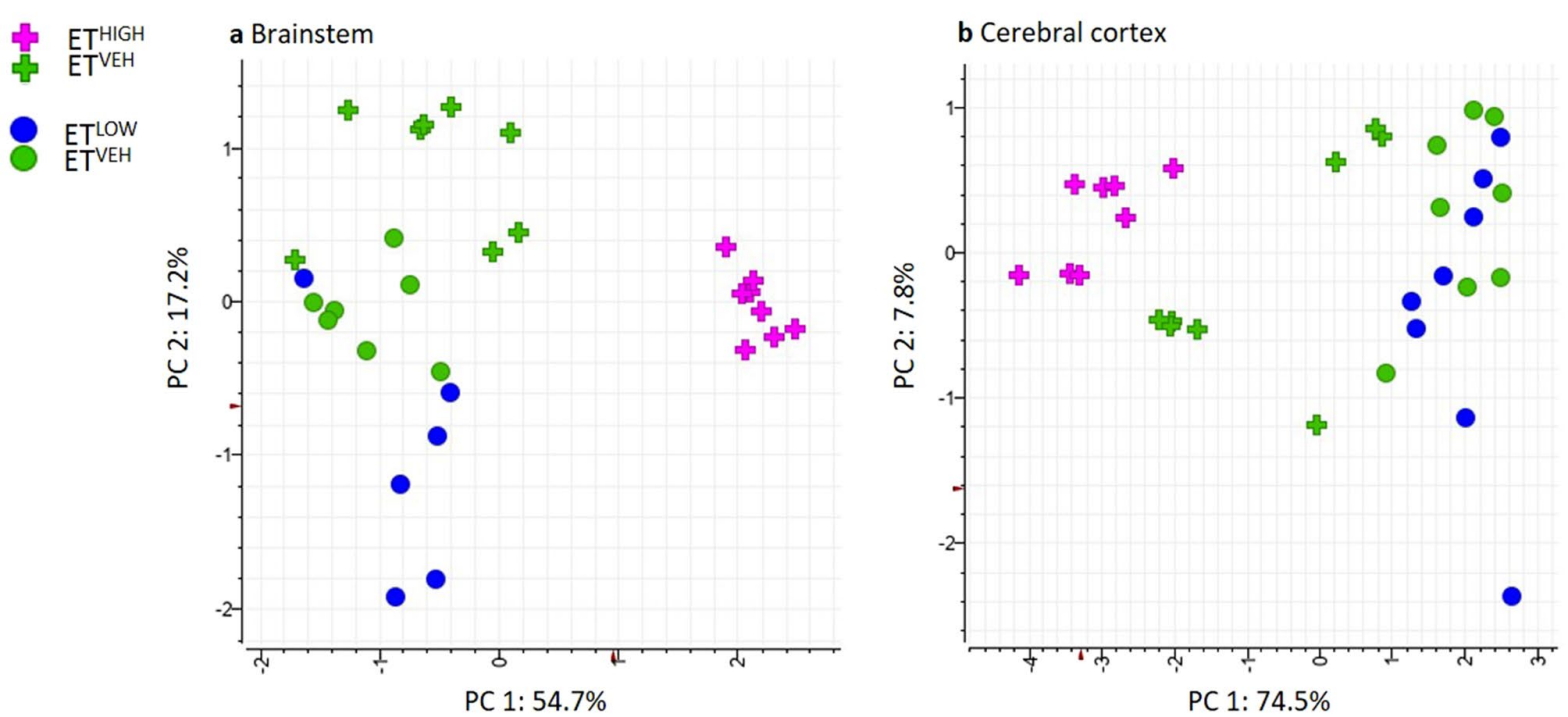
PCA scores plot of significant features generated from N-Way ANOVA of (**a**) brainstem (Q < 0.05) acquired from ESI+ UHPLC-HRMS of aqueous tissue extracts, representing classes ET^HIGH^ (pink; n = 8), ET^LOW^ (blue; n = 6) and ET^VEH^ (green; n = 8) and (**b**) cerebral cortex (Q < 0.01) acquired from ESI+ UHPLC-HRMS of aqueous tissue extracts, representing classes ET^HIGH^ (pink; n = 8), ET^LOW^ (blue; n = 8) and ET^VEH^ (green; n = 8).

A total of 3096 features were extracted from all brain tissue samples. Prior to statistical analysis, PCA plots of individual brain regions were generated to assess the reproducibility of the Pooled Biological Quality Control (PBQC) samples (Supplementary Fig. S1) for intensity drifts along the batch. Data dimensionality reduction was achieved using N-Way ANOVA on each of the brain tissue regions based on the treatment effect. The Benjamini-Hochberg (BH) correction criteria was used to adjust the significance (*p* value) of each of the variables, and the subsequent adjusted *p* value is referred to as a Q-value. A data subset with features satisfying Q-value < 0.05 were represented by 19 significant features in the brainstem. PCA plots performed on the dataset (n = 32) resulted in separation between classes brainstem ET^HIGH^ and ET^VEH^ across Eigenrow (herein Principal Component, PC) 1. A PCA plot generated for the brainstem represented 72% of the total variance (Fig. 3a). Most metabolic variation occurred between classes ET^HIGH^ and ET^VEH^ (54.7%, PC 1) compared to ET^LOW^ and ET^VEH^ (17.2%, PC 2). An orthogonal projection to latent structure discriminant analysis (OPLS-DA) model was applied to full datasets of the brainstem comparing ET^HIGH^ and ET^LOW^ treatments to ET^VEH^. The OPLS-DA scores plot revealed discrimination between treatment groups compared to controls in the brainstem (Supplementary Fig. S2). However, evaluation of model performance indicated poor predictive performance and goodness of fit for ET^HIGH^ vs ET^VEH^ (Q^2^ = 0.150, R^2^Y = 0.755, R^2^X = 0.057) and ET^LOW^ versus ET^VEH^ (Q^2^ = 0.325, R^2^Y = 0.771, R^2^X = 0.078). The overall models were not significant, indicated by 100 different model permutations for ET^HIGH^ vs ET^VEH^ (*p* value = Q^2^: *p* = 0.18 and R^2^Y: *p* = 0.64) and ET^LOW^ vs ET^VEH^ (*p* value = Q^2^: *p* < 0.01 and R^2^Y: *p* = 0.48).

A data subset with features satisfying a more stringent Q-value (Q < 0.01) cut-off was applied to the N-Way ANOVA results of the cerebral cortex, to allow discrimination of treatment effects in the PCA model. The cerebral cortex region showed dysregulation of 26 metabolites with a generated PCA plot representing 82.3% of the total variance (Fig. 3b). Only the ET^HIGH^ and ET^VEH^ (74.5%, PC 1) treatments could be discriminated in the PCA model. An orthogonal projection to latent structure discriminant analysis (OPLS-DA) model was applied to full datasets of the cerebral cortex comparing ET^HIGH^ and ET^LOW^ treatments with ET^VEH^. The OPLS-DA scores plot revealed discrimination between treatment groups compared to controls in the cerebral cortex (Supplementary Fig. S3). However, evaluation of model performance indicated poor predictive performance and goodness of fit for ET^HIGH^ vs ET^VEH^ (Q^2^ = 0.137, R^2^Y = 0.690, R^2^X = 0.058) and ET^LOW^ vs ET^VEH^ (Q^2^ =  − 0.032, R^2^Y = 0.744, R^2^X = 0.058). The overall models were not significant indicated by 100 different model permutations for ET^HIGH^ vs ET^VEH^ (*p* value = Q^2^: *p* = 0.12 and R^2^Y: *p* = 0.56) and ET^LOW^ vs ET^VEH^ (*p* value = Q^2^: *p* = 0.13 and R^2^Y: *p* = 0.83).

The cerebral cortex and brainstem regions shared 3 significantly dysregulated metabolites, these include a sterol lipid (Table 1) and 2 unknown compounds (Table 2).

**Table 1:** Summary of identified metabolites altered in the brain tissue in mice treated with ergotamine compared to vehicle control.

Identity	T_R_ (min)	Mass (m/z) [M + H]^+^	Molecular formula	Mass Error (ppm)	Tissue	ET^HIGH^ versus ET^VEH^		ET^LOW^ versus ET^VEH^		N-Way ANOVA		
						Effect size	*	Effect size	*	Q-Value (BH adjusted *p* value)	MS^2^ and MS^3^ ions	Metabolite Level
2-arachidonylglycerol	11.29	379.2835	C_23_H_38_O_4_	− 2.0536	brainstem	1/3.59	↓	1/1.79	↓	0.014	287.2375, 269.2271, 203.1798	2
Epinephrine	2.96	184.0966	C_9_H_13_NO_3_	− 1.1178	brainstem	1.13	↑	1/1.57	↓	0.039	166.0867, 120.0808, 124.0759	1
Pantetheine 4’-phosphate	2.94	359.1032	C_11_H_23_N_2_O_7_PS	− 4.3505	brainstem	1.03	↑	1/1.81	↓	0.039	–	3
Sterol lipid	8.72	415.2109	C_24_H_30_O_6_	− 1.5281	brainstem	7.36	↑	1.206	↑	3.23 × 10^−6^	303.1205, 264.1001	4
					cerebral cortex	3.47	↑	1/1.89	↓	1.99 × 10^−3^		
Adenylosuccinate	2.45	464.0808	C_14_H_18_N_5_O_11_P	− 1.1775	cerebral cortex	1.94	↑	1.43	↑	0.002	252.0728, 162.0774, 97.0288	2
Isobutyryl-L-carnitine	3.36	232.1540	C_11_H_21_NO_4_	− 1.3549	cerebral cortex	1.79	↓	1.27	↑	0.002	173.0814, 85.0286	2
S-3-oxodecanoyl cysteamine	7.06	246.1517	C_12_H_23_NO_2_S	− 2.2079	cerebral cortex	2.84	↑	1.25	↓	0.004	229.1263, 183.1204	2

*↑ = Up-regulated, ↓ = Down-regulated.

**Table 2:** Summary of significant unknown metabolites altered in the brain (cerebral cortex and brainstem) in ergotamine treated mice compared to control.

Group	T_R_ (min)	Mass (m/z) [M + H]^+^	Tissue	ET^HIGH^ versus ET^VEH^		ET^LOW^ versus ET^VEH^		N-Way ANOVA
				Effect size	*	Effect size	*	Q-Value (BH adjusted *p* value)
1	8.27	623.3156	brainstem	1/70.85	↓	1/1.27	↓	0.014
2	2.48	86.09672	brainstem	1/1.00	↓	1/1.34	↓	0.039
3	4.96	199.0939	brainstem	9.81	↑	–	–	0.023
4	1.99	267.0585	brainstem	1.20	↑	1.43	↑	0.039
5	10.18	292.2992	brainstem	1/3.03	↓	1/1.41	↓	0.036
6	2.02	308.5818	brainstem	1.32	↑	1/1.52	↓	0.039
7	8.04	387.1794	brainstem	–	–	1/4.20	↓	3.23 × 10^−6^
8	12.5	387.1972	brainstem	1.50	↑	1/7.24	↓	0.006
9	2.39	408.1484	brainstem	1.13	↑	1/1.26	↓	0.039
10	8.04	425.1353	brainstem	–	–	1/4.59	↓	6.34 × 10^−5^
			cerebral cortex	–	–	1/4.76	–	1.11 × 10^−5^
11	2.26	437.1332	brainstem	1.48	↑	1/1.44	↓	0.023
12	2.39	559.1500	brainstem	1.31	↑	1/2.15	↓	0.025
13	5.39	910.849	brainstem	1/1.40	↑	2.93	↑	0.039
14	1.18	258.1096	cerebral cortex	1/1.33	↓	1.04	↑	2.51 × 10^−4^
15	1.28	269.0875	cerebral cortex	1.71	↑	1/1.59	↓	5.05 × 10^−4^
16	2.39	269.0876	cerebral cortex	1.70	↑	1/1.31	↓	6.84 × 10^−4^
17	2.39	291.0695	cerebral cortex	1.45	↑	1/1.21	↓	5.05 × 10^−4^
18	16.7	123.0553	cerebral cortex	1/4.53	↓	1.22	↑	7.11 × 10^−4^
19	2.39	137.0456	cerebral cortex	1.70	↑	1/1.28	↓	5.05 × 10^−4^
20	1.82	159.0276	cerebral cortex	1.82	↑	1/1.41	↓	8.93 × 10^−4^
21	2.55	162.0581	cerebral cortex	2.73	↑	1/1.27	↓	6.88 × 10^−3^
22	4.99	204.1050	cerebral cortex	3.07	↑	1/1.31	↓	5.05 × 10^−4^
23	1.35	218.1384	cerebral cortex	1/2.10	↓	1.15	↑	2.19 × 10^−4^
24	2.61	218.1384	cerebral cortex	1/1.86	↓	1.19	↑	5.05 × 10^−4^
25	7.06	246.1517	cerebral cortex	2.84	↑	1/1.25	↓	4.37 × 10^−3^
26	1.25	276.1185	cerebral cortex	2.01	↑	1/1.40	↓	6.87 × 10^−3^
27	1.28	307.0434	cerebral cortex	1.75	↑	1/1.44	↓	5.05 × 10^−4^
28	9.63	315.1949	cerebral cortex	7.93	↑	1/1.37	↓	8.93 × 10^−4^
29	2.39	353.0399	cerebral cortex	2.14	↑	1/1.52	↓	1.99 × 10^−3^
30	2.39	369.0123	cerebral cortex	1.67	↑	1/1.30	↓	4.37 × 10^−3^
31	2.45	387.9961	cerebral cortex	1.97	↑	1/1.47	↓	1.99 × 10^−3^
32	1.28	464.0806	cerebral cortex	1.68	↑	1/1.51	↓	9.31 × 10^−3^
33	2.45	502.0274	cerebral cortex	2.09	↑	1/1.40	↓	9.95 × 10^−4^
34	8.04	409.1614	brainstem	–	–	1/4.20	↓	5.34 × 10^−7^
			cerebral cortex	–	–	1/4.20	↓	1.51 × 10^−9^

*↑ = Up-regulated, ↓ = Down-regulated.

N-Way ANOVA of the treatments ET^HIGH^, ET^LOW^ and ET^VEH^ in the thalamus and brainstem region showed no significant impact at Q < 0.05.

Together these data demonstrate that ergotamine treated (ET^HIGH^ and ET^LOW^) compared to ET^VEH^ animals show significant metabolic variation only in the brainstem and cerebral cortex regions of the brain. PCA plots of the significant (19 metabolites; Q-value < 0.05) metabolites in the brainstem region revealed a dose related effect, with clear separation between ET^HIGH^ and ET^LOW^. However, in the cerebral cortex region PCA of the significant metabolites (26 metabolites; Q-value < 0.01) showed that ET^LOW^ clustered with the ET^VEH^, indicating that only ET^HIGH^ had central effects.

### Identification of significant features

**Figure 4:**
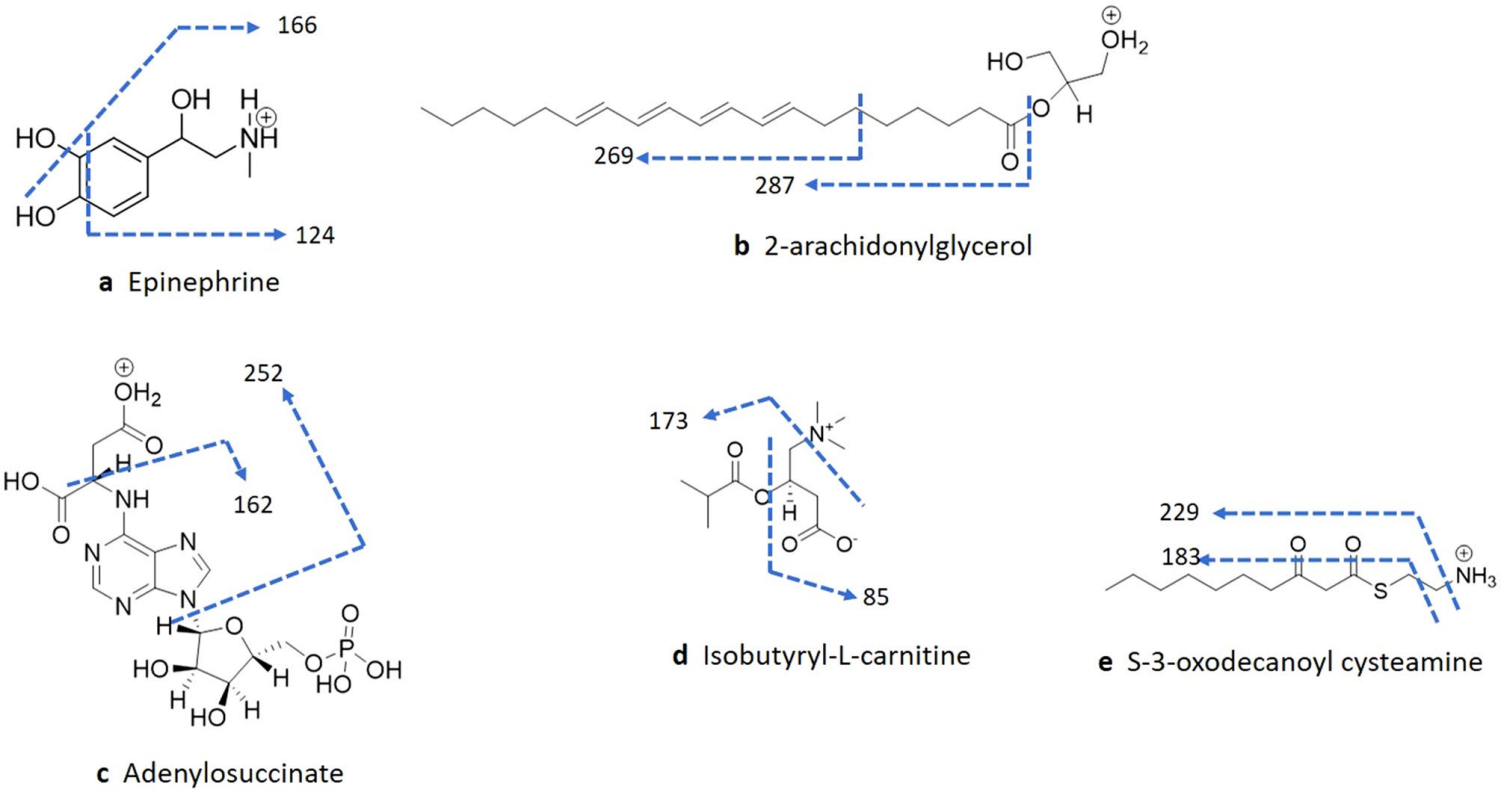
MS^n^ fragmentation confirm the identity of compounds differentially expressed in the brainstem and cerebral cortex regions of the brain.

Identification of metabolites impacted in the brainstem and cerebral cortex of animals exposed to ergotamine 1 h post treatment was investigated. For this, accurate masses were compared to the Human Metabolome Database (HMDB). Identification of 7 of the 45 differentially expressed metabolites was confirmed using either MS or MS^n^ fragmentation of the parent ion (Table 1, Fig. 4). There were 5 metabolites that achieved Level 2 identification and above (as defined by the Metabolomics Standards Initiative^25,26^); these include 2-arachidonylglycerol (Q-Value = 0.0386), epinephrine (Q-Value = 0.0386), adenylosuccinate (Q-Value = 0.0020), isobutyryl-L-carnitine (Q-Value = 0.0020) and S-3-oxodecanoyl cysteamine (Q-Value = 0.0004). MS^2^ fragmentation data of epinephrine and 2-arachidonylglycerol were consistent with those in the MzCloud database.

To confirm identity of the compounds, targeted MS^2^ and MS^3^ was performed on the 7 analytes (Supplementary Figs. S4–S8).

The catecholamine, epinephrine eluted at 2.96 min ([M + H]^+^, m/z 184.0966) and the MS^2^ mass spectrum gave rise to the intense ion m/z 166.0867 indicating deoxygenation. Subsequent demethylation of the N-CH_3_ and loss of remaining hydroxyl groups resulted in the less intense ion m/z 120.0808. Further MS^3^ of the most intense MS^2^ ion (m/z 166.0867) resulted in fragmentation of the aromatic ring (m/z 124.0759), confirming the presence of the compound (Fig. 4a).

The monoacylglycerol, 2-arachidonylglycerol (2-AG) eluted at 11.29 min (m/z 379.2835), MS^2^ fragmentation led to loss of the glycerol moiety m/z 287.2375. The MS^2^ ion m/z 287.2375 fragmentation resulted in deoxygenation and dehydrogenation (m/z 269.2271) prior to loss of the alkyloxy chain and (m/z 203.1798). The elution time and fragmentation pattern confirmed the identification of the compound (Fig. 4b).

Adenylosuccinate eluted at 2.45 min (m/z 464.0808) and MS^2^ fragmentation of the ion resulted in loss of the dehydroxylated tetrahydrofuran phosphate moiety (m/z 252.0728). The smaller fragments m/z 162.0774 and m/z 97.0288 represented the purine ring attached to the secondary amine group and a dehydrogenated methyl hydroxy tetrahydrofuran ring, respectively (Fig. 4c).

Isobutyryl-L-carnitine eluted at 3.36 min (m/z 232.1540). MS^2^ fragmentation led to fragmentation of the tertiary amine, m/z 173.0814, subsequent MS^3^ of the ion resulted in loss of the isobutyric acid group, m/z 85.0286 (Fig. 4d).

S-3-oxodecanoyl cysteamine eluted at 7.06 min (m/z 246.1517), and MS^2^ resulted in loss of the amine (m/z 229.1263) and subsequent MS^3^ of the ion represented the alkyl sulfane group (m/z 183.1204) (Fig. 4e).

The predicted molecular formula for m/z 415.2109 (C_24_H_30_O_6_) together with the fragmentation pattern and retention time (8.72 min), strongly suggest that the group belongs to the sterol lipid class of compounds (Level 4 identification).

The retention time 2.94 min and accurate mass (m/z 359.1032) in addition to a HMDB match suggest Level 3 identification of the compound pantetheine 4’-phosphate.

There are another 34 unknown metabolites that were also significantly altered in the brain (Fig. 3), and the accurate mass and retention time are listed in Table 2.

Receiver-operating characteristic (ROC) curves were used to evaluate the specificity and sensitivity of disrupted metabolites in the brainstem and cerebral cortex exposed to ET^HIGH^ compared to ET^VEH^. Sensitivity and 1-specificity corresponded to the true positive rate and false positive rate, respectively^27^. Areas under the curve (AUCs) have been applied as useful indices for discriminating the diagnostic values of features. If the AUC is greater than 0.7, the features are regarded as useful biomarkers^28–31^. AUC values greater than 0.7 are considered to be sensitive and specific. Using this criteria, 8 biomarkers in the brainstem (Supplementary Table S1) and 6 biomarkers in the cerebral cortex (Supplementary Table S2) were identified in ET^HIGH^ when compared to ET^VEH^. AUC results demonstrated that predictive models could be generated to determine ergotamine exposure in brain tissue of animals.

**Figure 5:**
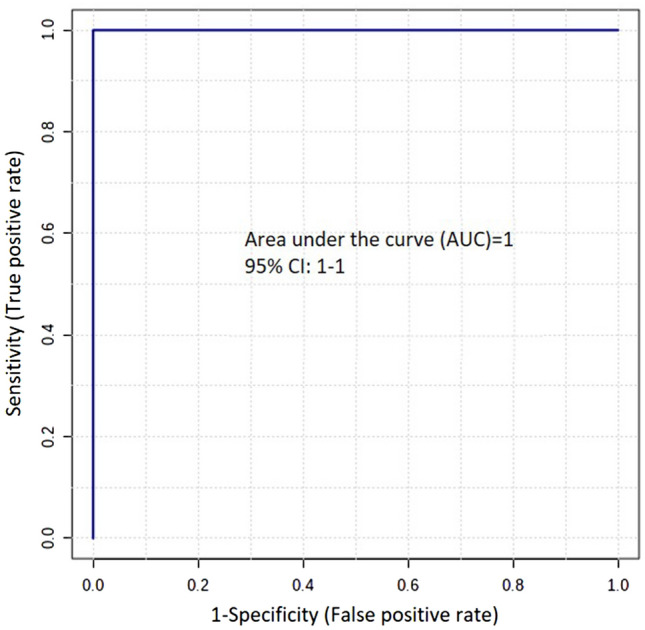
ROC curve of 2-arachidonylglycerol showing AUC—1.0 and sensitivity and specificity at a 95% confidence interval of 1–1.

To evaluate the predictive performance of sub-clinical exposure (ET^LOW^) to ergotamine, a combination of biomarkers for ET^HIGH^ was tested and linear support vector machine (SVM) was applied. Based on the prediction performance of ET^LOW^, 2-arachidonylglycerol was selected to build a classification model for the brainstem region. The ROC curve generated from the model resulted in an AUC of 1.0 and 1–1 at 95% confidence interval with 50% sample hold-out (Fig. 5). The cross-validation accuracy of the model showed good prediction performance of 0.88 based on 100 cross validations. The predictive performance of the model was found to be significant with 100 permutations (*p* = 0.010). The reliability of the model was tested on ET^LOW^ brain regions. The SVM model predicted with 67% (4 out of 6 replicates of ET^LOW^). These results indicate that the SVM model based on biomarkers confirms the importance of the metabolite 2-arachidonylglycerol in mild and severe cases of ergotamine intoxication in the brainstem.

## Discussion

This study investigated the mode and site of action of ergot alkaloids using ESI+ LCMS metabolite profiling to determine the effect of ergotamine exposure on brain metabolism in male C57Bl/6J mice at 1 h post treatment. Our results demonstrate that ergotamine elicits region specific effects in the cerebral cortex and brainstem, but not the cerebellum and thalamus. Ergotamine treated mice at high doses exerted the greatest metabolic variation in the brainstem where clear dose-related effects were also observed in the metabolite 2-AG. This is further corroborated by the detection of ergotamine in this region as previously reported in these same mice^7^. Recently, the clinical signs presenting in animal models exposed to ergotamine have been attributed to physiological effects in both blood pressure and heart rate that are regulated by the autonomic nervous system of the brainstem^7^. However, the physiological profile in these same mice were not affected in a dose dependent manner. This may be due to the receptors mediating the effect are reaching a physiological threshold^32^.

Ergotism in humans and other mammals results in behavioural effects and motor incapacitation which is associated with central nervous stimulation^33–37^. Our findings suggest that only intoxication at high concentrations of ergotamine effects the cerebral cortex metabolome significantly. This is in agreement with early studies that have shown that only very high doses of ergotamine result in behavioural disorders and motor incoordination in animals, including rodents^33^.

Non-detectable levels of ergotamine in the cerebral cortices^7^ suggest that the resulting behavioural effects could likely occur via excitation of afferent fibres entering the cerebral cortex from the neuromodulatory or cranial nerve nuclei of the brainstem.

Metabolomic profiling identified 45 metabolites significantly altered in the brain by ergotamine, that may be responsible for exerting a major effect in the brain at 1 h post treatment. Seven of these were identified using either MS or MS^n^ fragmentation of the parent ion.

The monoacylglycerol 2-AG is a key intermediate in multiple lipid metabolic pathways, and an endogenous endocannabinoid responsible for important physiological pathways such as locomotor activity, stress and anxiety^38^. A reduction of 2-AG in the brainstems of ergotamine treated mice relative to vehicle controls was found in this study. Decreased levels of 2-AG is linked to anxiety-like behaviour^39^, which is further supported by the behavioural phenotype elicited in mice exposed to high-levels of ergotamine^33^. The effect size of 2-AG compared to ET^VEH^ in this study decreased in a dose-dependent manner. Thus, 2-AG is shown to be a key metabolite for ergot alkaloid toxicity and its importance is further demonstrated by its ability to predict mild and severe cases of ergotamine intoxication in the ROC curve analysis.

The physiological phenotype of animals exposed to ergotamine is characterised by vasoconstriction which is likely to be responsible for other symptoms including increased blood pressure as well as cardiovascular and respiratory effects^7,9^. Perturbation in epinephrine neurotransmitter levels is associated with vasoconstriction and also likely to disrupt blood pressure and heart rate^40^ similar to those observed in ergotamine intoxicated animals. Ergotamine exposed animals suffer from gastrointestinal^41^ and cerebral vessel dilation^42^ all of which are associated with alterations in epinephrine levels^40^ mediated primarily through α-adrenergic receptors^33,43^. Epinephrine is synthesized in the brainstem in the medulla oblongata region and is involved in regulation of physiological functions of the sympathetic nervous system^44^. Epinephrine neurons in the brain, particularly the brain stem and hypothalamus regions, are involved in regulation of body temperature, reflex bradycardia as well as motor performance^45^.

Furthermore, variation in the synthesis of the metabolite pantetheine 4’-phosphate, a key intermediate in the biosynthesis of Coenzyme A (CoA) can lead to disruption in metabolic systems^46,47^ as CoA plays a key role in carbohydrate, lipid and amino acid metabolism^46,47^.

Together these findings support the hypothesis that the disrupted levels of epinephrine in ergot alkaloid-intoxicated animals show a complex interaction with biogenic receptors, primarily an α-adrenergic mediated response. Furthermore, psychoactive effects present in the behavioural phenotype can be attributed to disruption of lipids, particularly 2-AG and downstream metabolic systems.

The cerebral cortex comprises regions that regulate emotion, cognition, language, memory, homeostasis as well as auditory and visual areas^48^. The perturbed metabolites at high doses of ergotamine in the cerebral cortices include the energy related metabolites isobutyryl-L-carnitine and S-3-oxodecanoyl cysteamine. Impairment in energy metabolism is known to occur as a consequence of stress and toxicity and typically in a pathological state, where there is increased demand for energy production. Furthermore, ergotamine exposed animals show increased levels of adenylosuccinate in the cerebral cortex. The accumulation of adenylosuccinate in bodily fluids occurs due to a deficiency of adenylosuccinase, the enzyme which catalyses the conversion of adenylosuccinate into AMP in the synthesis of purine nucleotides^49^. The metabolic defect which results in a deficiency of adenylosuccinase can occasion mental impairment, ranging from a slight delay to severe retardation^49^.

These studies indicate that behavioural effects and motor incoordination exhibited by ergot alkaloid intoxicated animals at high doses of the toxin could be attributed to the metabolic disruption in the cerebral cortex.

## Conclusion

This is the first study exploring the effects of ergot alkaloid toxicity on the brain metabolome in a rodent model. Metabolomics profiling identified key differences in epinephrine, 2-AG and panthetheine 4′-phosphate in the brainstem region, which we suggest are linked to the physiological effects exhibited by animals exposed to the toxin. Furthermore, pathophysiological processes elicited only in response to high doses of the toxin in the cerebral cortex, could contribute to the behavioural dysfunction and motor incoordination exhibited in animals ingesting ergot alkaloids. Understanding the cause and biochemical consequences of toxicoses provide insight into potential treatments or pharmacological uses. Thus, future studies should be undertaken to determine the identification of the unknown metabolites in this study.

## Materials and methods

### Toxins

Ergotamine (98% pure) was purchased from Novachem Pty Ltd, (B-MYC3600-5). Ergotamine was administered by intraperitoneal (ip) injection in the vehicle carrier 1% lactic acid (neat lactic acid, Sigma Aldrich, diluted with ultrapure distilled water, Invitrogen).

Ergotamine was administered at 0.025 mg/kg bwt (ET^LOW^) and 0.05 mg/kg bwt (ET^HIGH^). These doses correspond to sub-clinical (low) and potent (high) doses that are calculated based on levels of feed intake of ruminants, concentrations in pasture and pharmacological aspects such as bioavailability of the toxin^8^. A negative control group was used for comparison of physiological and behavioural observations for each cohort, vehicle control (ET^VEH^) treated with 1% lactic acid via intraperitoneal (ip) injection.

### Animal studies

All animal studies were approved by the La Trobe University Animal Ethics Committee (Protocol number 18–21) and were conducted in accordance with the Australian Code of Practice for the Care and Use of Animals for Scientific Purposes set out by the National Health and Medical Research Council of Australia. The research study was conducted in compliance with the ARRIVE (Animal Research: Reporting of In Vivo Experiments) guidelines^50^. Animal experimental procedures were as previously described^7^ and provided here in brief. The mice were housed in groups of two to four during the experimental period in individually ventilated cages (Tecniplast, Buguggiate, Italy) with standard pellet food and water available ad libitum. Ambient temperature of housing and testing rooms was 21 ± 2 °C and mice were housed under a 12-h light–dark cycle (lights on at 7am). A total of 48 male C57Bl/6J mice 8–12 weeks old were sourced from a breeding colony at the Walter and Eliza Hall Institute of Medical Research, Melbourne, Victoria. Animals were allowed to acclimatise to the facility conditions for a minimum of one week prior to the experiment phase. All animals underwent rotarod testing to examine effects of intoxication on movement and tail plethysmography to observe vasomotor effects^7^.

### Metabolite extraction

Tissues of mice exposed to low (ET^LOW^; 0.025 mg/kg bwt, n = 6) or high (ET^HIGH^; 0.05 mg/kg bwt, n = 8) doses of ergotamine were collected at 1 h post-treatment and compared to vehicle injected controls (ET^VEH^; n = 8) by metabolomic analysis^7^. Animals were euthanized by cervical dislocation and brain tissues dissected into cerebral cortex, thalamus, cerebellum and brainstem were harvested. Samples were snap-frozen in liquid nitrogen before storage at − 80 °C for metabolomics analysis.

Frozen samples of thalamus and cerebral cortex were transferred into 4 mL polycarbonate tubes with 3/8" stainless steel grinding balls and kept frozen in liquid nitrogen. Sample tubes were placed into pre-frozen 24 well cryo-blocks on the Geno/Grinder 2010 (SPEX Sample Prep, Metuchen, NJ, USA) and the tissues were homogenised at 1700 rpm for 1–5 min^7,51^. The fine powder was stored at − 80 °C until weighed. Frozen cerebellum and brainstem tissues were hand-ground in a chilled mortar and pestle with liquid nitrogen. The fine powder was stored at – 80 °C until weighed.

Cerebral cortex, thalamus, cerebellum and brainstem (20–23 mg) samples were each weighed in 2 mL microcentrifuge tubes (Eppendorf SafeLock). Tissue samples were extracted using a 4:1 (v/v) MeOH/H_2_O mono-phasic methanol extraction^7^. Briefly, 500 μL chilled 80% methanol was added to frozen tissue powder and vortex-mixed (15 s). Samples were sonicated on ice for 10 min, incubated at room temperature for a further 10 min, then centrifuged for 5 min at 10,000 rpm (9503 × *g*). The extraction was repeated and supernatant pooled to give a final volume of 1 mL and stored at − 80 °C.

A volume of 50 μL was transferred into HPLC vials containing inserts ready for LCMS analysis of polar metabolites. Each extract of a tissue type (2 µl of each) was combined to generate a pooled biological quality control (PBQC) sample, which was used to monitor analytical reproducibility. The remaining supernatant was evaporated under a stream of nitrogen, re-constituted in 100 µL of 4:1 (v/v) MeOH/H_2_O, and transferred to HPLC vials containing 200 µL inserts for quantitative LCMS analysis.

### Liquid chromatography-mass spectrometry (LCMS) analysis

Metabolite profiling was performed on the Vanquish Ultra-High Performance Liquid Chromatography (UHPLC) system (Thermo Fisher Scientific, Bremen) with a binary pump, autosampler and temperature-controlled column compartment coupled with a QExactive (QE) Plus mass spectrometer (Thermo Fisher, Waltham, MA, USA; Thermo, Bremen, Germany)^7^ for MS and MS^2^ analysis or a Thermo Scientific LTQ Orbitrap Velos ion trap MS system (Thermo Scientific, Waltham, MA, USA; Bremen, Germany) for MS^3^ analysis. Metabolites eluted on a Thermo Fisher Scientific Hypersil Gold 1.9 µm, 100 mm × 2.1 mm column. The initial conditions were 2% B before initiating a linear gradient to 100% B over 11 min, and this was maintained for 4 min before returning to the initial gradient conditions at a flow of 0.3 mL/min (total run time of 20 min).

The Thermo Fisher QExactive Plus mass spectrometer was used to acquire MS data and set at positive mode over a mass range of 70–1200 amu with resolution set at 17,000. Nitrogen was used as the sheath, auxiliary and sweep gas at a flow rate of 28, 15 and 4 L/min, respectively, and spray voltage was set at 3600 V (positive mode)^52^. Samples were randomized, and blanks (80% methanol) were injected every five samples. A PBQC was run every 10 samples. Prior to data acquisition, the system was calibrated with Pierce LTQ Velos ESI Positive and Negative Ion Calibration Solution (Thermo Fisher Scientific). Mass spectrometry data was acquired using Thermo Xcalibur V. 2.1 (Thermo Fisher Scientific Inc., USA). For MS^2^ analysis, data were acquired in full-scan MS/data-dependent MS^2^ (ddMS^2^) mode for selected samples. MS cycles were composed of 1 Full MS and up to 5 ddMS^2^. Ions within the inclusion list detected in the full MS survey scan (intensity threshold 4.0 × 10^4^) triggered a MS^2^ event at the peak apex with an isolation window of 0.4 m/z. A 2.0 s delay was required for the same ion to trigger a new MS^2^ event (dynamic exclusion). Full MS scans were acquired from m/z 300 to 1200 with a resolution of 70,000 (full width at half maximum, FWHM, at m/z 200); automatic gain control (AGC) target was 3 × 10^6^; maximum injection time (IT) 200 ms. Scans (ddMS^2^) were acquired at a resolution of 17,500, the AGC target was 1 × 10^5^, and the maximum IT was 50 ms. Ions were fragmented with stepped collision energy (20, 40 and 60%).

Compound fragmentation using MS^2^ and MS^3^ analysis was performed using a Thermo Scientific LTQ Orbitrap Velos ion trap MS system (Thermo Scientific, Waltham, MA, USA; Bremen, Germany), with a heated electrospray ionisation (ESI) source. Data-dependent MS^2^ and MS^3^ spectra was acquired on target ions and selected samples with normalised collision energy of 35 V and an ion max time of 50 microseconds. Source heater temperature was maintained at 350 °C and the heated capillary was maintained at 320 °C. The sheath, auxiliary and sweep gases was 40, 15 and 8 units respectively, for ESI+ mode. Source voltage was set to 4.2 kV (positive) and with a capillary voltage of − 70 V. Sample injection volumes was 5 µL. Prior to data acquisition, the system was calibrated with Pierce LTQ Velos ESI Positive and Negative Ion Calibration Solution (Thermo Scientific). Spectra was inspected in Thermo Xcalibur Qual Browser v.2.3.26 (Thermo Fisher Scientific).

Prior to data analysis, individual samples were checked for column pressures, variations of PBQC and retention times for instrument stability along the batch.

### Data processing and statistical analyses

The data files obtained following LCMS analyses were processed in the Refiner MS module of Genedata Expressionist 12.0 with the following parameters: (1) chromatogram chemical noise subtraction with removal of peaks with less than 4 scans, chromatogram smoothing using moving average estimator over 5 scans, and 70% quantile over 151 scans for noise subtraction, (2) intensity thresholding using a clipping method and a threshold of 100, (3) selection of positive mode data only, (4) chromatogram RT alignment using a pairwise alignment based tree and a maximum RT shift of 2 min, (5) chromatogram peak detection using a 5 scans summation window, a minimum peak size of 0.1 min, a maximum merge distance of 0.05 Da, a boundary merge strategy, a maximum gap/peak ratio of 70% with moving average smoothing over 10 scans for peak RT splitting (6) chromatogram isotope clustering using RT and m/z tolerance of 0.05 min and 0.05 Dalton respectively with a maximum charge of 2, (7) adduct detection using mainly M + H and allowable adducts (M + 2H, M + K, M + Na, M-H_2_O + H) and (8) singleton filter for removal of peaks that do not belong to a cluster, resulting in a total of 3096 number of features (Supplementary Fig. S9).

Statistical analyses were performed using the Analyst module of Genedata Expressionist 12.0. Principal component analyses (PCA) were performed to identify tissue and treatment differences. Overlay of the PBQC and samples allowed for the validation of the high-quality dataset by ensuring that RT variation, mass error and sensitivity changes throughout the run were consistent. Statistical significances between treatments within tissue types were assessed by N-Way ANOVA and sorted according to BH Q-Value. Levels of significance for brainstem (Q < 0.05) and cerebral cortex (Q < 0.01) were applied and PCA performed on significant metabolites.

An OPLSDA model was applied to the brainstem and cerebral cortex datasets using MetaboAnalyst 3.0^53^ Prior to multivariate analysis missing value imputation was applied and features with > 50% missing values were removed and remaining missing values replaced by 1/5 of the minimum positive value for each feature. Subsequently, the data were log transformed and mean centered to achieve normality and homoscedasticity. A prediction model was also developed on MetaboAnalyst 3.0 to discriminate effects between control and treatment groups using ROC curve model. The significant features were evaluated and identified for each brain tissue for ergotamine high dose treatment. The ROC area under the curve (AUC) analyses for tissues brainstem and cerebral cortex were ranked based on AUC values (Supplementary Tables S1 and S2). All potential biomarker candidates were subjected to linear SVM (support vector machine) to establish a statistical prediction model.

Analyte identification of significant metabolites was performed by searching experimental MS^1^ data through the following databases: Human Metabolome DataBase (HMDB) (http://hmdb.ca); ChemSpider (http://chemspider.com); and Lipid Maps®(http://www.lipidmaps.org); . MS^2^ data was searched on MzCloud (http://mzcloud.org) and MetFragment®(http://ipb-halle.github.io/MetFrag/).

## Supplementary Information

Supplementary Information.

## Data Availability

The datasets generated during the current study are available from the corresponding author on reasonable request.
