# Supplementary material for: Effects of ergotamine on the central nervous system using untargeted metabolomics analysis in a mouse model
Source: Sci Rep. 2021 Oct 1;11:19542. doi: 10.1038/s41598-021-98870-4 (PMC8486802; doi:10.1038/s41598-021-98870-4)
Supplement: Supplementary file 1 — Supplementary Information. [file 41598_2021_98870_MOESM1_ESM.docx]

**Effects of ergotamine on the central nervous system using untargeted metabolomics analysis in a mouse model**

**Supplementary information**

Priyanka Reddy, Delphine Vincent, Joanne Hemsworth, Vilnis Ezernieks, Kathryn Guthridge, German C Spangenberg, Simone J Rochfort

**Supplementary Table S1.** ROC curve analysis of biomarkers for ET^HIGH^ exposed animals (brainstem).

| **Metabolite** | **Brainstem: ET^HIGH^ vs ET^VEH^** | | | |
| --- | --- | --- | --- | --- |
|  | AUCs | T-tests | Log2FC | KM Cluster |
| sterol lipid | 1.0 | 2.72 x 10^-6^ | 2.70 | 2 |
| 2-arachidonylglycerol | 1.0 | 1.4 x 10^-5^ | -2.01 | 5 |
| Group # 6* | 0.875 | 0.0090 | 0.041 | 5 |
| Group # 5* | 0.859 | 0.070 | -1.89 | 4 |
| tianeptine | 0.766 | 0.101 | 0.20 | 5 |
| Group # 4* | 0.750 | 0.069 | 0.25 | 5 |
| epinephrine | 0.719 | 0.155 | 0.21 | 5 |
| pantatheine 4’-phosphate | 0.703 | 0.125 | -0.22 | 5 |

*Refer to Table 2.

**Supplementary Table S2.** ROC curve analysis of biomarkers for ET^HIGH^ exposed animals (cerebral cortex).

| **Metabolite** | **Cerebral cortex: ET^HIGH^ vs ET^VEH^** | | | |
| --- | --- | --- | --- | --- |
|  | AUCs | T-tests | Log2FC | KM Cluster |
| sterol lipid | 1.0 | 1.64 x 10^-4^ | 1.45 | 2 |
| Group # 9* | 0.906 | 0.013 | 1.449 | 1 |
| tianeptine | 0.891 | 0.013 | 0.88 | 1 |
| Group # 6* | 0.797 | 0.062 | 0.34 | 5 |
| Group # 5* | 0.781 | 0.107 | -1.82 | 3 |
| piperidine | 0.750 | 0.102 | 0.19 | 5 |

* Refer to Table 2.


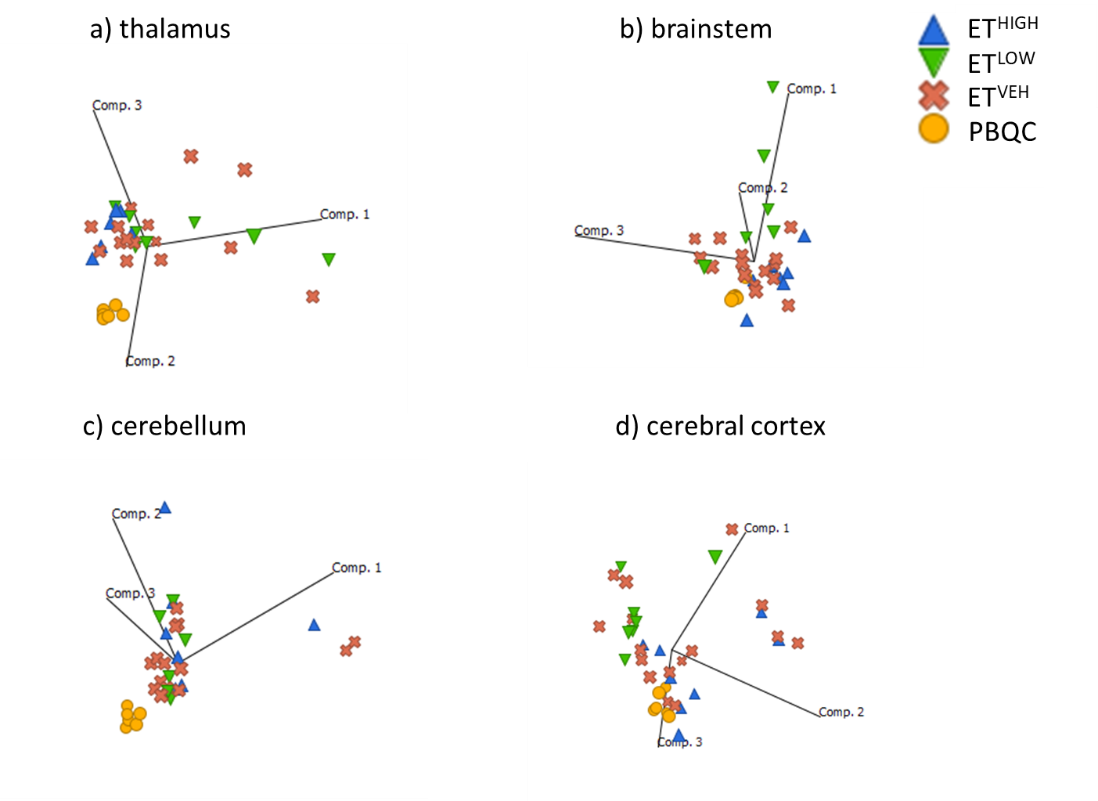
**Supplementary Figure S1**. PCA scores plot of ESI+ UHPLC-HRMS data acquired from the aqueous extracts of individual brain tissue: (a) thalamus, (b) brainstem (c) cerebellum, (d) cerebral cortex of treatments: ET^HIGH^ (blue; 0.05 mg/kg; n=8), ET^LOW^ (green; 0.025 mg/kg; n=6) and ET^VEH^ (orange; 1% lactic acid; n=8) demonstrating reproducibility of Pooled Biological Quality Control (yellow; PBQC) samples.

**
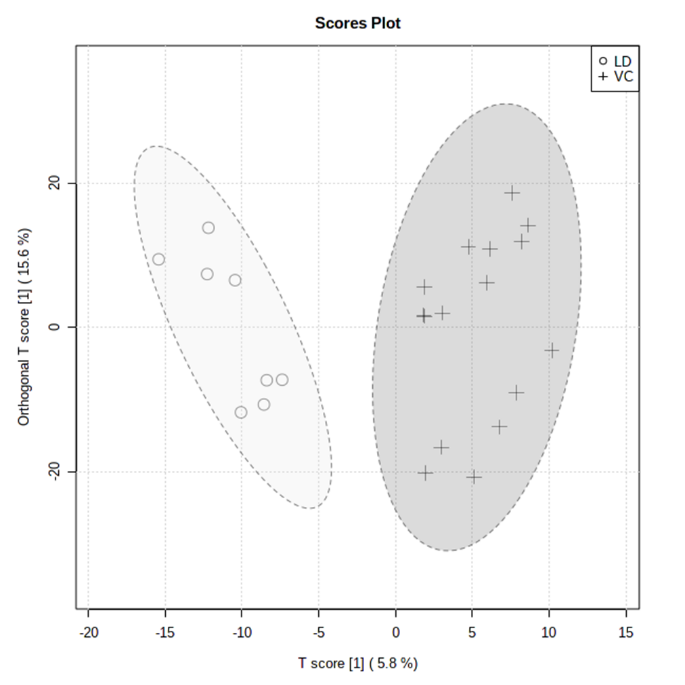
**
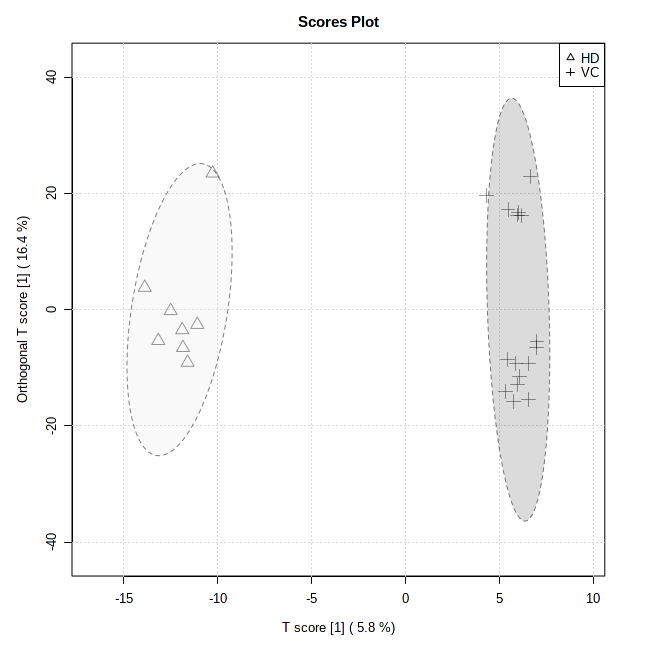
**
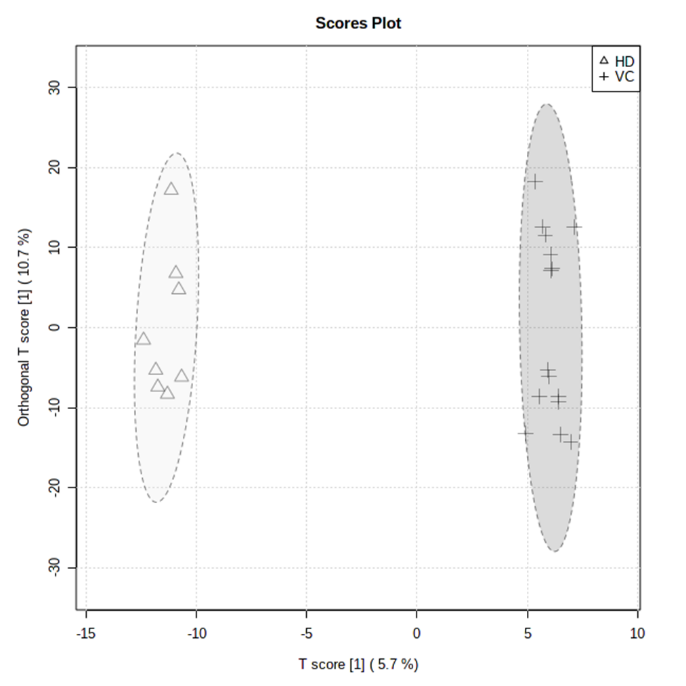
**
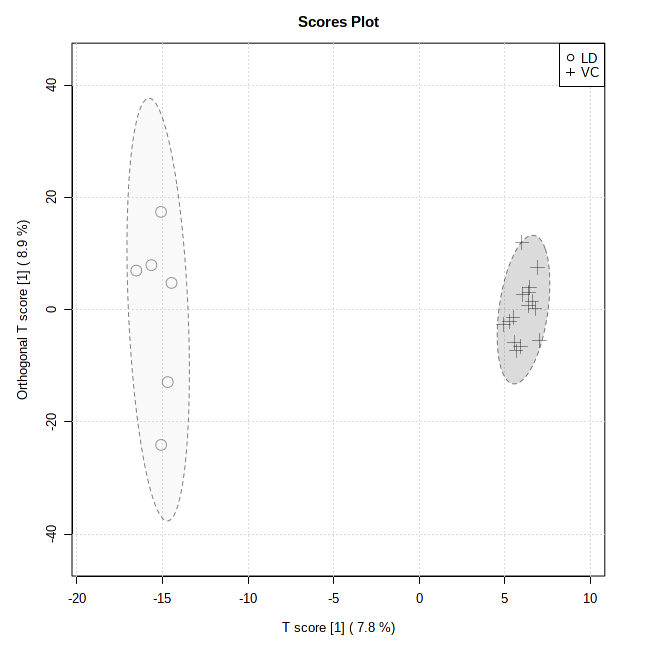
**Supplementary Figure S2.** OPLS-DA scores plots of brainstem tissues: (A) OPLS-DA scores plot with associated 95% confidence ellipses demonstrating distinction between ET^HIGH^ and ET^VEH^ with poor predictive performance and goodness of fit (Q^2^=0.150, R^2^Y =0.755 R^2^X = 0.057), the overall model was not significant indicated by a permutation test (p-value = Q^2^: p = 0.18 and R^2^Y: p =0.64); (B) OPLS-DA scores plot with associated 95% confidence ellipses demonstrating distinction between ET^LOW^ and ET^VEH^ with poor predictive performance and goodness of fit (Q^2^=0.325, R^2^Y =0.771, R^2^X = 0.078), the model was not significant indicated by 100 different model permutations (p-value = Q^2^: p < 0.01 and R^2^Y: p =0.48).

(B)

(A)

(B)

(A)

**Supplementary Figure S3.** OPLS-DA scores plots of cerebral cortex tissues: (A) OPLS-DA scores plot with associated 95% confidence ellipses demonstrating distinction between ET^HIGH^ and ET^VEH^ with poor predictive performance and goodness of fit (Q^2^=0.137, R^2^Y =0.690, R^2^X = 0.058), the overall model was not significant indicated by 100 different model permutations (p-value = Q^2^: p = 0.12 and R^2^Y: p =0.56); (B) OPLS-DA scores plot with associated 95% confidence ellipses demonstrating distinction between ET^LOW^ and ET^VEH^ with poor predictive performance and goodness of fit (Q^2^=-0.032, R^2^Y =0.744, R^2^X = 0.058), the model was not significant indicated by 100 different model permutations (p-value = Q^2^: p = 0.13 and R^2^Y: p =0.83).


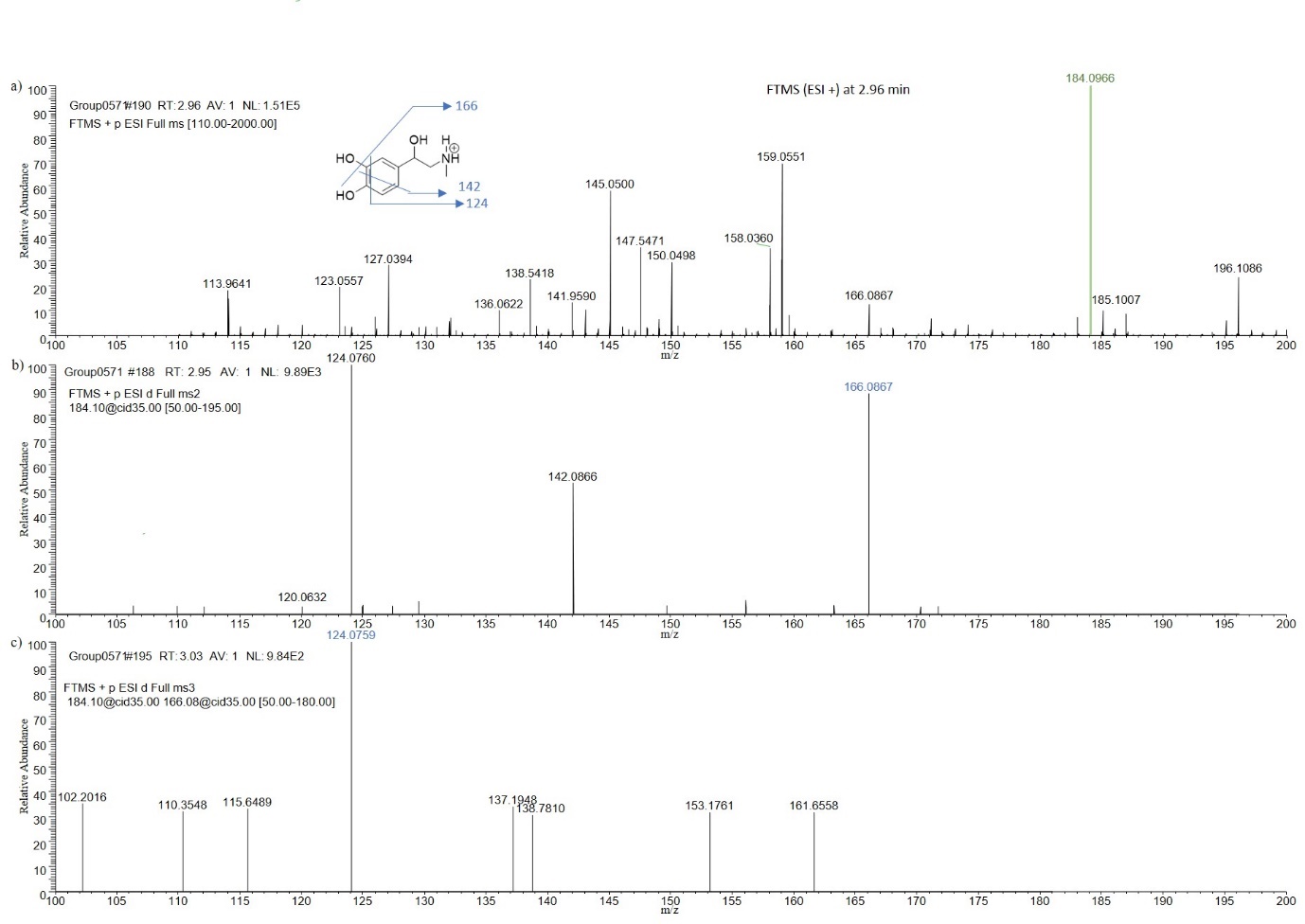
**Supplementary Figure S4**. Mass spectra of epinephrine in positive ion mode (LC-ESI-FTMS). (a) Precursor MS^1^ spectrum with an observed ion at m/z 184.0974 (Δ -1.12 ppm) at 2.96 min as well as (b) MS^2^ and (c) MS^3^ fragmentation spectra confirms identity of epinephrine in the brain.


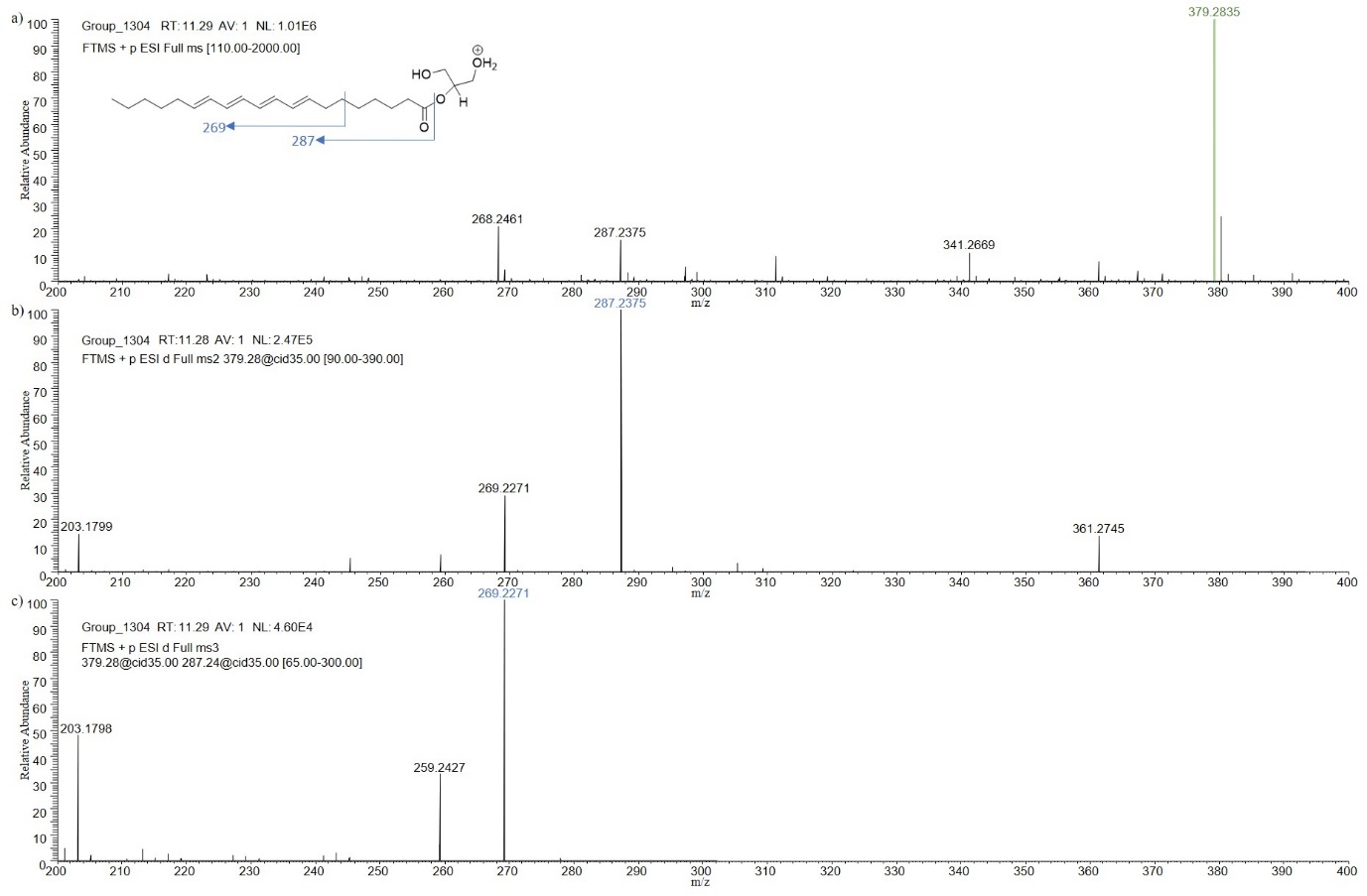
**Supplementary Figure S5.** Mass spectra of 2-arachidonylglycerol in positive ion mode (LC-ESI-FTMS). (a) Precursor MS^1^ spectrum with an observed ion at m/z 379.2851 (Δ -2.05 ppm) at 11.28 min as well as (b) MS^2^ and (c) MS^3^ fragmentation spectra confirms identity of 2-AG in the brain.


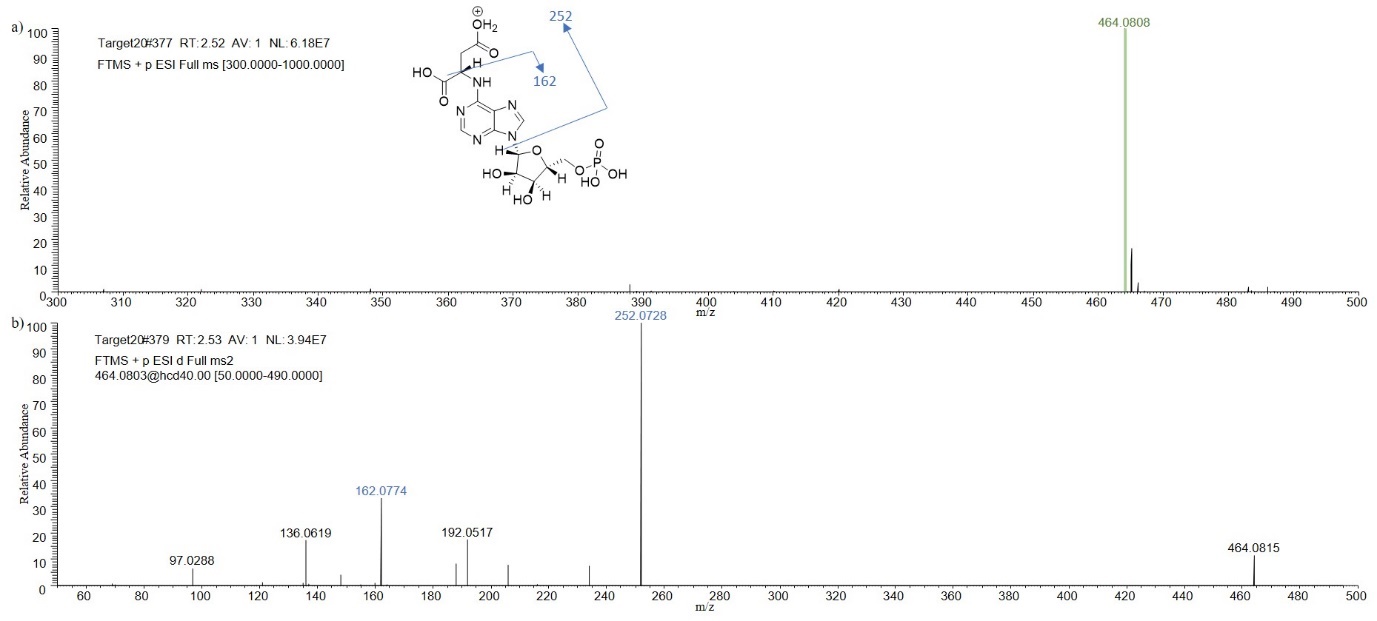


**Supplementary Figure S6.** Mass spectra of adenylosuccinate in positive ion mode (LC-ESI-FTMS). (a) Precursor MS^1^ spectrum with an observed ion at m/z 464.0817 (Δ -1.18 ppm) at 2.52 min and the (b) MS^2^ fragmentation spectrum confirms identity of adenylsuccinic acid in the brain.


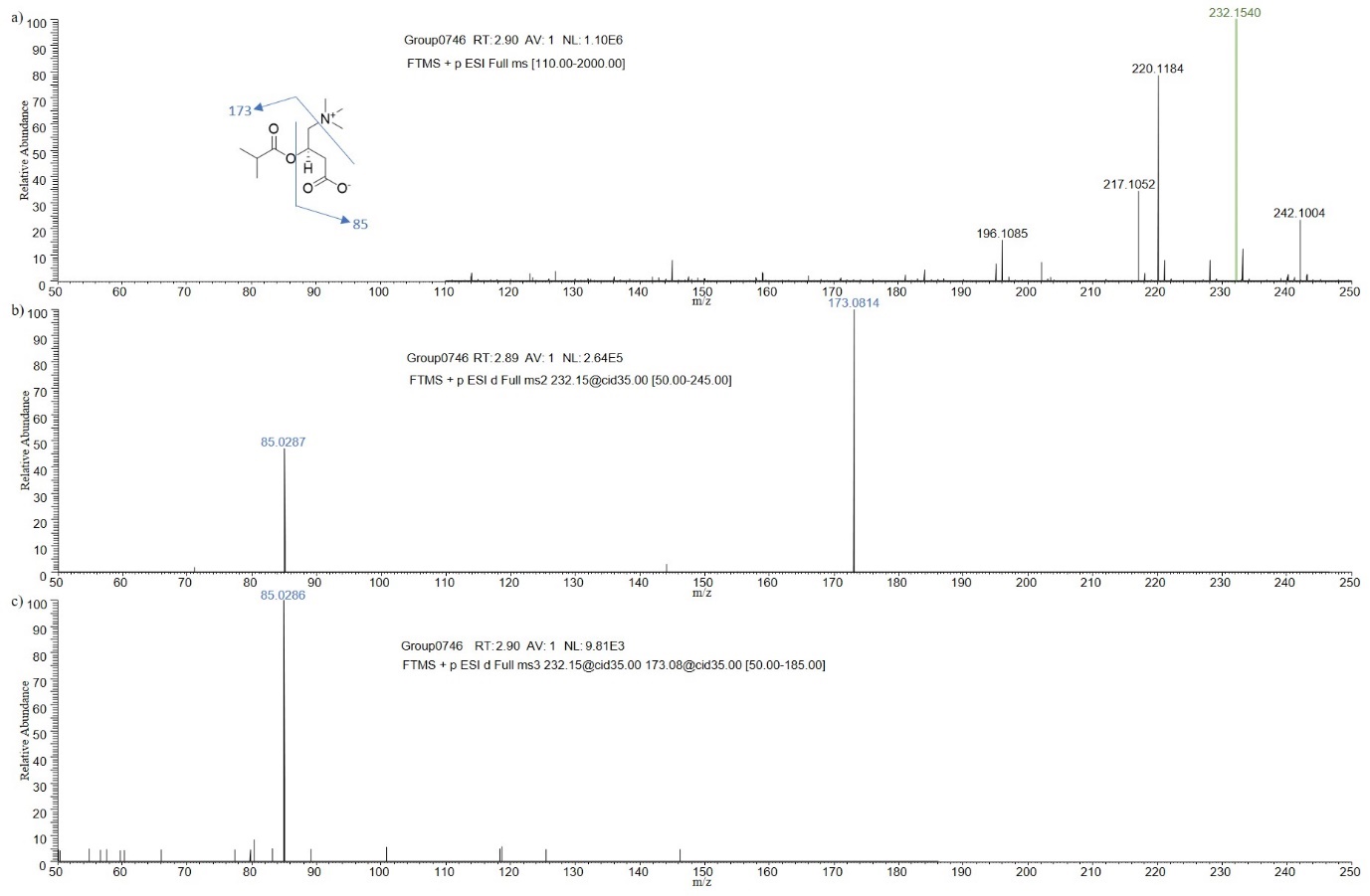


**Supplementary Figure S7.** Mass spectra of isobutyryl-L-carnitine in positive ion mode (LC-ESI-FTMS). (a) Precursor MS^1^ spectrum with an observed ion at m/z 232.1548 (Δ -1.35 ppm) at 2.90 min as well as (b) MS^2^ and (c) MS^3^ fragmentation spectra confirms identity of isobutyryl-L-carnitine in the brain.


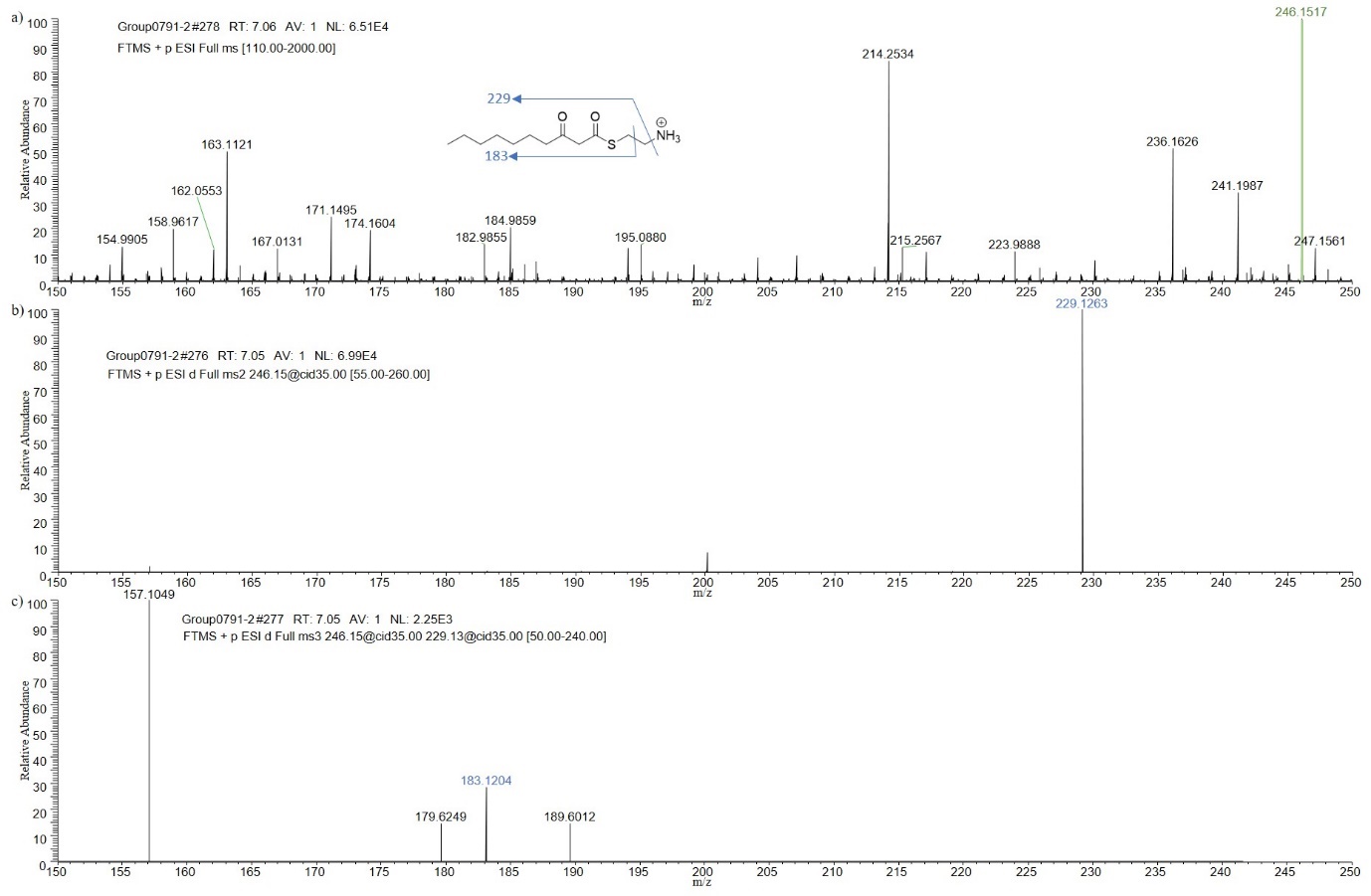
**Supplementary Figure S8.** Mass spectra of S-3-oxodecanoyl cysteamine in positive ion mode (LC-ESI-FTMS). (a) Precursor MS^1^ spectrum with an observed ion at m/z 246.15288 (Δ -2.21 ppm) at 7.06 min as well as (b) MS^2^ and (c) MS^3^ fragmentation spectra confirmed identity of S-3-oxodecanoyl cysteamine in the brain.


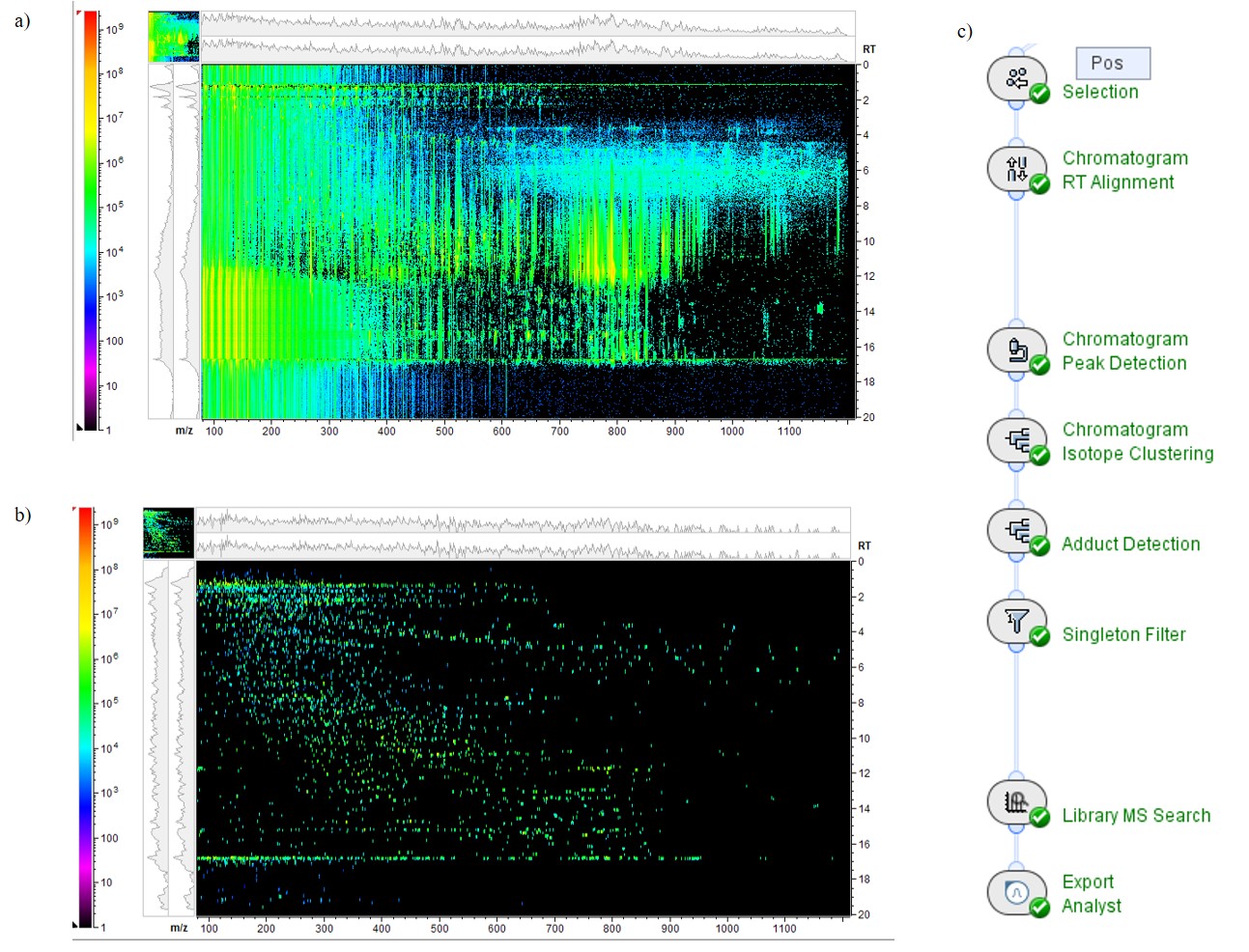


**Supplementary Figure S9.** LCMS (+ESI positive) data files imported to Genedata Expressionist (a) were processed in the Refiner MS module (b) with nodes that allow for chromatogram chemical noise subtraction; chromatogram RT alignment; peak detection; chromatogram isotope clustering; adduct detection and removal of single ions followed by (c) library search through database Human Metabolome DataBase (HMDB).
